# Intersecting Inequities in COVID-19 Vaccination: A Discourse Analysis of Information Use and Decision-Making Among Ethnically Diverse Parents in Canada

**DOI:** 10.1007/s40615-024-01940-2

**Published:** 2024-02-26

**Authors:** Emmanuel A. Marfo, Terra Manca, Eunah Cha, Laura Aylsworth, S. Michelle Driedger, Samantha B. Meyer, Catherine Pelletier, Ève Dubé, Shannon E. MacDonald

**Affiliations:** 1 Faculty of Nursing, University of Alberta, Edmonton, AB Canada; 2 Faculty of Health Disciplines, Athabasca University, Athabasca, AB Canada; 3 Department of Community Health Sciences, Rady Faculty of Health Sciences, University of Manitoba, Winnipeg, MB Canada; 4 School of Public Health Sciences, University of Waterloo, Waterloo, ON Canada; 5 Centre de Recherche du CHU de Quebec- Université Laval, Quebec City, QC Canada; 6 Department of Anthropology, Université Laval, Quebec City, QC Canada

**Keywords:** Ethnically diverse parents, COVID-19 vaccination, Information, Health inequities

## Abstract

**Background:**

Little is known about how intersecting social privilege and disadvantage contribute to inequities in COVID-19 information use and vaccine access. This study explored how social inequities intersect to shape access to and use of COVID-19 information and vaccines among parents in Canada.

**Methods:**

We conducted semi-structured interviews on COVID-19 vaccination information use with ethnically diverse parents of children ages 11 to 18 years from April to August 2022. We purposefully invited parents from respondents to a national online survey to ensure representation across diverse intersecting social identities. Five researchers coded transcripts in NVivo using a discourse analysis approach informed by intersectionality. Our analysis focused on use of vaccine information and intersecting privileges and oppressions, including identifying with equity-denied group(s).

**Results:**

Interview participants (*N* = 48) identified as ethnically diverse non-Indigenous (*n* = 40) and Indigenous (*n* = 8) Peoples from seven Canadian provinces. Racialized minority or Indigenous participants reflected on historical and contemporary events of racism from government and medical institutions as barriers to trust and access to COVID-19 information, vaccines, and the Canadian healthcare system. Participants with privileged social locations showed greater comfort in resisting public health measures. Despite the urgency to receive COVID-19 vaccines, information gaps and transportation barriers delayed vaccination among some participants living with chronic medical conditions.

**Conclusion:**

Historicization of colonialism and ongoing events of racism are a major barrier to trusting public health information. Fostering partnerships with trusted leaders and/or healthcare workers from racialized communities may help rebuild trust. Healthcare systems need to continuously implement strategies to restore trust with Indigenous and racialized populations.

## Introduction

There are many barriers to accessing vaccinations and relevant information for those with intersecting social inequalities across diverse ethnicities, employment, incomes, geographic location, language, and education [1–5]. For example, medical mistreatment and harm by the health system create barriers to trusting authoritative information sources, especially for those previously harmed by state systems [6–8]. Health systems must demonstrate trustworthiness and avoid blaming underserved populations for distrust [9].

Previous studies reported little understanding of how intersections of social privilege and disadvantage contribute to inequities in COVID-19 information use and vaccine access among diverse parents in Canada [3, 10]. Parents with certain privileges, such as those who can afford alternative treatments, may refuse to vaccinate [11], especially if they perceive their families as healthy and less vulnerable to COVID-19. Yet, parents’ concerns about vaccination for their children often differ from their concerns for themselves [12]. For example, parents may consider age, sex, chronic medical conditions, and immediate and long-term safety concerns when making child(ren)’s vaccination decisions. In addition, social disruption and concerns about threats to perceived freedoms from public health measures (e.g., mask and vaccine mandates) may shape information choice and engagement, consequently influencing parental vaccine decisions [13, 14].

There is a need for research to understand how to ensure credible information is accessible and trustworthy to communities and individuals with prior and/or ongoing experiences of medical mistreatment, racism, and intersecting systemic inequities. An intersectional exploration of COVID-19 vaccination can assist in examining and responding to the relevant complex and interlocking factors that shape information access and use [5, 15]. Yet, a recent scoping review reported limited use of intersectionality in vaccination research in Canada [16]. Cha et al. found that even in instances where intersectionality concepts were used in vaccination research, individual-level characteristics were mostly studied [16], neglecting the intersections of structural and systemic influences on vaccination inequities.

In this study, we explored the question: how do social inequities intersect to shape parents’ access and use of COVID-19 information and vaccines for themselves and their children? Insights from this study may inform tailored public health policy and interventions in Canada and international contexts with similar inequality indices such as disease risk, vaccination, and the healthcare system. This important study will also contribute to interventions intended to mitigate unintentional harms of health policies that neglect the voices of communities with intersecting identities during and after global health crises [17].

### Study Context: Canada and Childhood COVID-19 Vaccination

Canada is a country predominantly governed by White settlers and descendants with institutions built on colonial legacies. Canada has a well-documented history of systemic discrimination, including mistreatment and racism in health policy, healthcare, and across most state agencies [18, 19]. There is an ongoing colonization of Indigenous Peoples (First Nations, Métis, and Inuit) through existing colonial legacies and institutionally- legitimized policies and practices [18, 20, 21]. Furthermore, like many multiethnic countries, racialized minorities are subjected to systemic racism and discrimination across intersecting social locations, including ethnicity, gender, age, language, social class, dis(ability), employment, geographical location, migration status, citizenship, and histories [18, 22]. These systemic factors influenced the COVID-19 vaccination rollout and access across Canadian provinces and territories.

In late 2020 and early 2021, there was a limited COVID-19 vaccine supply and COVID-19 vaccines were initially rolled out to higher risk populations. Vaccine supply improved throughout mid-2021, and vaccine access was extended to the general population, including children [23]. At the onset of this study (April 2022), COVID-19 vaccines were in adequate supply in Canada, and individuals 5 years and older were eligible for vaccination.

Canadian and international research shows that parents consider different factors and may make different vaccination decisions for their children than themselves [24, 25]. Prior research found parents’ vaccine decisions are shaped by their socio-historical context, values, understandings of vaccination, and beliefs (which may include conspiracies) [26]. Factors considered important to parents for childhood vaccination decisions include concerns about the safety and novelty of COVID-19 vaccines [27, 28], the age of their child(ren) [28], and the possibility of long-term adverse effects on their child(ren) [29].

In early 2022, parents considered these factors in the context of heightened public discourse about COVID-19 vaccination. The politicization of COVID-19 vaccination and fear of potential stigmatization may have created discomfort for parents to express vaccine concerns or raise questions about child(ren)s’ COVID-19 vaccination. Social disruption and organized opposition to COVID-19 vaccine mandates (e.g., “Freedom Convoy Movement”) [30, 31] shaped public discourse about COVID-19 vaccines on online media, which incorporated notable racist, hate speech, and discriminatory discourses [32, 33]). Parents who sought information online also did so in the context of an “infodemic” [34], in which an overwhelming amount of accurate and inaccurate information was in circulation, requiring effort to locate credible COVID-19 vaccine information, reject problematic information, and navigate through misinformation.

### Theoretical Approaches: Intersectionality and Discourse Analysis

Intersectional analyses focus on concurrent interlocking and interplaying of social locations such as race, gender, employment, education, nationality, language and more and/or relevant power relations (e.g., racism, colonialism, and patriarchy) [35, 36]. Informed by earlier feminist and critical scholarship, including critical race theory, intersectionality shows that human experiences are complex, irreducible, and inseparable [35, 37]. Intersectionality provides a framework to assess how social and political processes, including policies and systems, reinforce domination, and subordination [36]. This approach provides insights into how multiple social locations/identities coexist to create unique pandemic experiences, information engagement, and vaccine decisions [38, 39].

Applying an intersectional approach allows us to examine the compounded individual, social, and systemic inequities in COVID-19 information and vaccination use and access among Canadian parents [40]. To facilitate an analysis of privileges and oppressions that mutually shaped COVID-19 information engagement and vaccine use [41], we applied a feminist critical discourse analysis (CDA) [42]. Feminist CDA is particularly useful for analyzing how systemic inequities are (re)produced and diffused through patterns of meanings, including human symbols, behaviors, language, and communication [42].

## Methods

### Overview

This article reports findings from one of four stages of a large multifaceted project, each applying a discourse analysis approach to investigate different aspects of vaccine information engagement and decision-making in ethnically diverse parents. For this objective, we conducted semi-structured interviews with parents who had one or more children aged 11–18 years old. Focusing on intersecting social locations, parents were selected by purposeful sampling.

The project team included researchers identifying with both privileged and equity-denied populations, some identifying with those more severely affected by the pandemic in Canada (i.e., racialized and Indigenous), with diverse expertise to facilitate an interdisciplinary approach informed by medical anthropology, nursing, sociology, public health, and health communications.

### Participant Recruitment

Participants were recruited from respondents to an online survey for the larger project, conducted by a Canadian polling firm [43] to explore respondents’ COVID-19 vaccine attitudes, decisions, and access. Survey respondents were 18 years or older and could answer an internet-based questionnaire in English or French (Canada’s two official languages). For this study, participation was restricted to respondents who identified as a parent or guardian to at least one child between the ages of 11 and 18. Of 6026 respondents to the national survey, 3402 consented to be contacted for follow-up interviews, of which 360 met our inclusion criteria. We prioritized inviting survey respondents whose survey responses did not suggest extremes of either strongly preferring or refusing COVID-19 vaccines. Participants were purposefully selected along the spectrum of vaccine hesitancy (e.g., undecided, preferring to delay vaccination) and across diverse intersecting social identities/locations (e.g., ethnic identities, province of residence, genders, migration/citizenship status, first language, income, education, and employment). We invited 142 survey respondents to complete an online consent form and schedule an interview. Subsequently, 48 parents completed an interview between April and August 2022.

### Data Collection

Five research team members conducted semi-structured interviews in English and French. The interview guide was informed by survey results and developed in consultation with a large diverse team of researchers. Interviews focused on participants’ perception of COVID-19 pandemic, factors considered when deciding on vaccination for their child(ren) and themselves, and engagement with (mis)information sources on COVID-19 vaccinations. We tailored the interview questions on the spot to further explore participants’ experiences and concerns, including health experiences with COVID-19 vaccines. Interviews were conducted via Zoom video conferencing (*n* = 47) or telephone (*n* = 1) and lasted 22 to 60 min. Participants received a $40 CAD gift card as compensation for their time. Interviews were audio-recorded with the participants’ consent and transcribed for analysis in NVivo software (QSR International, Burlington, MA).

### Data Analysis

We coded transcripts for evidence of intersecting privileges and oppressions facilitated by a feminist CDA approach. The feminist CDA approach offered insights into coding discourses about public health messaging, COVID-19 vaccination, individual responsibility and privileges, and racism. After coding, we organized codes into relevant themes of intersecting inequities that commonly emerged in the interview data. We were attentive to relationships among socio-political factors (e.g., historical experiences, identities, beliefs, values, and concerns) contributing to information access and use. Our use of interviews and intersectionality theory further allowed us to attend to participants’ identities and locations, including the understandings and meanings they attached to them [36]. To lessen participation burden among study participants [44], who have already engaged in online surveys and interviews amid global crises and COVID-19 related social disruption, we did not conduct member checking. We recorded our personal reflections in memos and regularly discussed emerging findings. We formed consensuses on coding during regular team meetings. Study participants were assigned pseudonyms to ensure anonymity.

## Results

### Study Participants

Interview participants (*N* = 48) reported having child(ren) aged 11 to 18 years (*N* = 82). Participants were diverse across ethnic and Indigenous identities with the largest group identifying as White (*n* = 14, see Table 1). The majority of participants resided in Ontario or Quebec (*n* = 29), identified as women (*n* = 31), participated in the study in English (*n* = 38), and had post-secondary education (*n* = 41). One parent identified as Two-Spirit. Most parents (*n* = 40) reported receiving two or more COVID-19 vaccine doses, while eight reported receiving no COVID-19 vaccine dose. As reported by the parents, 69 children had received at least one dose, while 13 received no doses (see Table 2). Throughout the results, we refer to participants’ ethnic identity using language they identified during the interview (e.g., South Asian, Filipina, Spanish, Jewish) (see Appendix, Table 3).

**Table 1:** Summary of participants’ characteristics (*N* = 48)

Characteristic	*n* (%)
Ethnicity	
Arab	2 (4.2**)**
Black	4 (8.3)
East Asian	8 (16.7)
Latin American	3 (6.3)
South Asian	8 (16.7)
Jewish	1 (2.1)
White	14 (29.2)
Indigenous	
First Nations	4 (8.3)
Métis	2 (4.2)
Indigenous (no details)	2 (4.2)
Age (years)	
< 39	11 (22.9)
40–49	22 (45.8)
50–59	14 (29.2)
> 60	1 (2.1)
Gender	
Woman	31 (64.6)
Man	16 (33.3)
Two-Spirit	1 (2.1)
Province	
Atlantic (PEI)	1 (2.1)
Eastern (ON, QC)	29 (60.4)
Western (AB, BC, SK, MB)	18 (37.5)
Interview language	
English	38 (79.2)
French	10 (20.8)

**Table 2:** COVID-19 vaccine decisions

*n* (%)	
Parents (*N* = 48)	
2 doses + booster	29 (60.4)
2 doses	11 (22.9)
1 dose	0 (0.0)
0 doses	8 (16.7)
Children 5–18 years (*N* = 82)	
2 doses + booster	10 (12.2)
2 doses	54 (65.9)
1 dose	5 (6.1)
0 doses	13 (15.9)

^*^Children over 5 years old were eligible for vaccination at the time of interview. Excludes children under 5 years old, one adult child over 18 years old, and one of an unknown age

Focusing on how intersecting social locations shaped parents’ COVID-19 information use and vaccination uptake, we applied a feminist CDA approach to investigate how coexisting inequities shaped COVID-19 information use and vaccine decisions among participants. Three major themes emerged from the analysis around how systemic inequities shaped (1) trust in information sources and differential responses to public health measures, including vaccination, (2) exposure to COVID-19 and access to COVID-19 vaccines through work, and (3) perceptions of the safety and necessity of COVID-19 vaccination for personal health conditions. Below, we provide a detailed report of the emerged themes and their related sub-theme(s).

- **Theme 1: Trust in Information Sources and Differential Responses to Public Health Measures, Including Vaccination**

Participants’ trust in, access to, and acceptance of COVID-19 information and vaccination was shaped by intersecting privileges and disadvantages. Some participants with privileged social locations showed greater comfort in resisting public health measures, often perceiving their families to be at lower risk of severe COVID-19 disease. Other participants discussed privileges and disadvantages specific to their workplace as shaping their vaccination decisions and perceived risk from COVID-19 disease.

- **Sub-theme 1: Systemic Racism, Trust, and Access**

Parents who identified as racialized minorities or Indigenous spoke about historical and contemporary racism from government and medical institutions as barriers to trust and access to COVID-19 information, vaccines, and the Canadian healthcare system. Rachel, who identified as Indigenous, explained:

[I]t was only two generations ago that our grandmothers were getting forcibly sterilized, [...] that our uncles were part of the malnutrition experiments on Vancouver Island and other places […]. This is history in our memory that [...] we have very valid concerns about how we’ve been treated by the medical system and by the government, […] that is a huge barrier for a lot of Indigenous People to get the shot. No there’s [...] still systemic racism right now going on with healthcare treatments for Indigenous People.

Similarly, participants who self-identified as racialized minorities mentioned barriers to trust and questioned the intentions behind COVID-19 vaccination. These concerns were informed by non-dominant discourse about systemic racism, including narratives of recent and historical medical mistreatment of participants’ ancestors. Leonard, who identified as Black, repeated a narrative based on popular discourses about the unethical and deceitful “Tuskegee Experiment”^1^ in the United States about how Black people were “injected with syphilis without their knowledge and consent.” Leonard connected such abhorrent medical racism to the perceived intended purpose of COVID-19 vaccination, “Those kind of things [pause] have caused me to really consider you know, is this [COVID-19 vaccination] an agenda by the government to remove my members from, target, the Black community?” Leonard continued, referring to the history of sterilization racism:

I know some rumours were spreading that you know this, [...] it’s like a birth control […] especially targeted Black uh Black women to suppress their […] fertility [...]. So that’s another concern that we had in the family.

Leonard was particularly concerned about whether the vaccine could negatively affect his daughters and if it may be administered differently to women and girls who are Black than those who are White. He contextualized these concerns with accounts of microaggressions that were consistent with racism that he experienced throughout the pandemic, showing these issues were contemporary, ongoing, and institutional.

Some participants reported experiences of being blamed for the pandemic due to racist discourses about COVID-19. Kasey who identified as Southern Chinese, recounted, “there have been several incidents where I have been called names […], or [others] have said things to me to indicate that […] China caused all these problems for us.” Likewise, Elias, who identified as East Asian, linked such experiences of racism, and blame to public discourse that privileges White citizenship, which he felt intensified during the pandemic. Elias stated, “even though we have stayed here in Canada for many years, there was animosity towards us, China and Asia, because COVID-19 originated from there […] this was not from the government but by the public.” Kasey and Elias’s experiences of blame and racism suggest feelings of being *Othered* due to systemic racism, which reproduces a racial hierarchy of belonging, privileging White Canadian citizens who often fail to recognize that “there’s still systemic racism right now.”

- **Sub-theme 2: Social Privilege and (Non)compliance with Public Health Measures**

Most participants reported intentions, if not desire, to follow recommended public health measures (e.g., physical distancing, isolation, vaccination). However, others rejected these measures or only followed them when mandated. Participants who resisted these measures often also identified with privileged groups (e.g., White, able-bodied, “healthy”), which were associated with less risk from COVID-19 disease and were less likely to experience systemic discrimination (e.g., racism, ableism). Antoine, who identified as a White Francophone, reported ease in following public health measures, “We followed the instructions. My boy had no choice. He had to do tele-study at home like all the kids. So, he came home […] I’m still lucky, I have a pretty big house” (translated from French). He perceived his family to be at low risk from COVID-19 and expressed disdain towards these measures. Antione attributed this perceived low risk to personal behaviors and physical health stating, “I am a healthy person. My spouse is a healthy person. So is my son. We’re healthy. My parents are healthy […] We have a good immune system […], I am not someone who likes to be vaccinated, I like to exercise. I like to eat well.” After refusing vaccination, Antoine relied on his privileged networks with pharmacists to illegitimately obtain vaccine passports for himself and his son so that he could access services without being vaccinated:

My wife works in the pharmaceutical field. We have lots of friends who work in that field. He [the pharmacist] says, 'You want a passport? Okay that's nice. We'll go through the motions. Come to the pharmacy, we'll say you took the dose,’ then you don't take it […] He just fills out the paperwork"

In contrast, many participants with close migration ties to resource-limited regions of the world expressed awareness of their privilege to access COVID-19 vaccines in Canada earlier than people in those regions. For instance, Neila who recently immigrated to Canada from Venezuela, spoke about how her decisions to get vaccinated, vaccinate her youngest child, and encourage her adult stepson to get vaccinated were motivated by the loss of friends in Venezuela, who had no opportunity to get vaccinated:

I think that is […] a great opportunity for us in Canada, or United States, or in Spain, [...] people have the opportunity for vaccine […]. They died, my two best friend [names] only reinforce […] the vaccination is positive. Only reinforce. I think, that is when I take the opportunity to take the vaccine.

Other migrant parents attributed the unawareness of relatively abundant freedoms in Canada as motivation to assert individual rights and resist public health measures. Gabriel, who identified as a Southeast Asian, spoke to cultural differences in perceptions of collective responsibility and individual freedom:

It is a privilege to live in the West and I do feel that we don’t appreciate what we have access to here and perhaps we have a little too much freedom. I mean the convoy here in Ottawa and what not […] People are so high on wanting to hold on to their rights but they don’t realize that in a way they almost have too many […]. I mean you have obligations and responsibilities to the people around you.

- **Theme 2: Exposure to COVID-19 and Access to COVID-19 Vaccines Through Work**

Participants’ descriptions of their employment, exposure to COVID-19, and access to vaccination demonstrated their social position at intersecting sites of disadvantage and/or privilege. Some participants, including healthcare and other essential workers, expressed concerns about the risk of exposure to COVID-19. Kasey, who identified as Chinese, shared her concerns about risk as an essential worker who was among those not prioritized for early vaccination:

Before the protocols were in place [...] we didn’t have shields on the counter [...] we didn’t have face masks and gloves, so there was concern, since we were serving people, there might be a higher risk of contracting COVID-19.

Kasey elaborated on the challenge of being pleasant to customers while feeling frustrated and angry with those who refused to wear a face mask. Combined with a lack of protective resources at her workplace early in the pandemic, she felt at higher risk of contracting COVID-19.

Other participants who were essential workers and lacked early access to vaccination reported additional challenges due to pandemic restrictions. For instance, Rachel, who identified as an Indigenous parent, reported seeing and experiencing abuse at work when enforcing public health mandates (e.g., mask-wearing):

Because the first time you’re called an [cuss words], or the first time you’re, you’re spit at, or sworn at, you know you take it, and then it happens again the next day. It happens again, four or five days later […] and each incident on their own is not, like that bad, but when it happens again and again and again, you’re constantly on your guard and you start […] it’s almost like being an abuse victim.

Rachel’s account shows increased risks of exposure to COVID-19 and to offensive behaviors and abuse from her clients.

Another complication with work exposure was identified by a participant who explained that public health authorities did not hold private companies accountable for protecting their employees’ health and implementing public health measures. Caleb, who identified as Jewish, explained that his employment in a private railway industry was essential and high exposure, but excluded from prioritization for early access to vaccination, despite repeated exposure to COVID-19:

We had outbreaks in our bunk houses all the time. Guys were showing up sick to work with COVID-19. Like, literally sick to work with COVID-19 infecting their coworkers, and no one in the press ever heard about it, no one.

Feeling deprioritized and at high risk of COVID-19 infection, Caleb said that he accessed a COVID-19 vaccine earlier than he was eligible to: “I just walked down there and said I worked in healthcare industry, even though I don't, and they gave me a shot of Pfizer.”

- **Theme 3: Perceptions of the Safety and Necessity of COVID-19 Vaccination for Personal Health Conditions**

Participants described the importance and safety of vaccination in relation to their own and their loved one’s health status. Those who perceived themselves to be in good health often spoke about personal health practices (e.g., exercise, diet) in addition to or instead of vaccination and other public health measures. Many participants, however, indicated that vaccination was more urgent, especially if they had underlying health conditions. Yet, some identified evidence gaps about COVID-19 vaccination among those with specific health conditions as a barrier to their vaccine acceptance.

- **Sub-theme 1: Perceived Autonomy to Protect Health**

Across privileged and disadvantaged social locations, participants who perceived COVID-19 vaccination as less necessary did not report having chronic health conditions and often reported what they considered to be health-promoting behaviors. Beth, who identified as a White single parent who was currently unemployed, discussed how individual behaviors can protect health instead of vaccination: “If you keep your immune system and body healthy if you feed yourself healthy and exercise, there is no need for that [vaccination].” Similarly, Leonard, who identified as Black and did not mention health conditions, discussed his personal lifestyle choices and health practices as contributing to his decision not to get boosted every year for the flu shot as well as his desire not to receive COVID-19 vaccine boosters. Instead, he focused on his health practices: “Lifestyle choices, and I don’t smoke, I don’t drink.”

Brenda, who identified as South American, shared her feelings of safety and personal engagement in following precautions to keep her safe during the pandemic. She implied confidence in her family’s precautionary measures:

We took our meds, we stayed home, we rested. We ate our chicken noodle soup. You know, we did the normal stuff you do whenever you get sick [....] I just took precautions, and yes, I followed the restrictions and everything else, but I wasn’t to the point that [...] I need to barricade myself in my home.

While Brenda and her family may have taken preventive health measures to avoid getting sick, she also elaborated on the privilege of a healthy immune system: “My kids are healthy. No medical conditions […] I would prefer their body to […] fight this off naturally.” This underlying health privilege allowed Brenda and her family to choose whether to receive or refuse the COVID-19 vaccine.

- **Sub-theme 2: Gaps in Evidence Specific to Health Conditions**

In contrast, many participants with chronic medical conditions mentioned challenges in accessing credible and relevant information on vaccine side effects specific to their health conditions. Some perceived vaccination as important but delayed the timing of their vaccination in the hopes that evidence associated with their condition would emerge. Mariane, a White Francophone living with compromised immunity, spoke about her search for credible information:

Then I was trying to find information; there was as much misinformation as there was real information […] because I have a lot of allergic reactions or I have side effect reactions to stuff […]. So, I have to be very careful [...]. That’s what I’m afraid of, when I start a new medication: Will I have a reaction? So that’s what I was afraid of at first. So, after that, having talked about it with my doctors when they knew how I react to things, they told me, ‘you’re going to be ok with this.’ (Translated from French).

Similarly, Shakira, who identified as Indigenous and a single parent with compromised immunity and a neurodiverse child, spoke to an additional responsibility to inform her vaccine decisions with information specific to her family’s health. To navigate this responsibility, Shakira found information about COVID-19 vaccination through trusted networks with similar health conditions. Referring to vaccine decision-making for her child, Shakira explained: “I asked [other parents] how their kids reacted with being autistic as well, if there was anything I could do at the doctors to make it more easy for him.”

Gaps in information related to vaccine side effects for individuals with chronic health conditions deterred Alice, who identified as White from vaccination:

I chose not to because when they first came out with the vaccine, I know the news had said [...] the people in charge of vaccines were saying that people with lots of health problems like diabetes and allergies should hold off getting them, until they know all the side effects of them. So, I decided not to do it because of those reasons, because I’m type two diabetic and I have severe allergies.

Mariane, Shakira, and Alice’s accounts highlight the challenges and expectations of some individuals with chronic medical conditions to locate relevant information and advocate for their care in Canada.

- **Sub-theme 3: Intersections of Health Conditions and Urgency to Vaccinate**

Some participants expressed urgency to get vaccinated because of their health status. Shakira was concerned that exposure to COVID-19 could kill her: “I build resistance to those medications, so they don’t work for me. So, for me to have gotten COVID pneumonia, I wouldn’t have survived that.” As the primary caregiver to her children who live with disabilities and did not wear masks at school, Shakira not only had concerns that her children might bring COVID back home, but the compounded responsibility of keeping herself safe to care for her children. Despite the urgency to get vaccinated and eligibility as First Nations, she was unable to access her first dose for 6 months due to a lack of transportation. Had she not been faced with such barriers, Shakira reports that: “For medical reasons, I would have got it sooner.”

Some parents feared potential adverse health outcomes of COVID-19 for their children with chronic medical conditions. Of these parents, even some who reported refusing routine vaccines for their children were motivated to vaccinate their children for protection against COVID-19. Anais, a single mother who identified as White and French speaker, discussed their decisions: “My daughter has epilepsy, so she can’t get fever. So that was a really big concern. So, when they came out with the childhood vaccination, she was the first one to go. I signed her up quickly” (translated from French). Anais’ concerns for her daughter’s safety were heightened by her work in a high-exposure environment and her daughter’s exposure to COVID-19 at school. Although Anais found public health measures unpleasant, she felt they were necessary to protect her high-risk daughter from getting severely sick with COVID-19. Her lingering concerns about vaccinating her children diminished after her own vaccination: “But once we, the adults were vaccinated, we saw that it was not so bad, finally. I was much less worried about giving it to the kids”.

## Discussion

### Principal Results

In this study, we investigated how social inequities intersect to shape information use and vaccine decision-making among diverse parents. The study findings align with prior research showing that systemic racism and intersecting inequities contributed to barriers to trusting and using credible COVID-19 information and vaccination among participants and their children [7, 45–48] and motivations to get vaccinated among those with health conditions [49]. Our findings add to the literature about how historicizing colonialism and recent events of racism in public discourse shape engagement in health information and vaccination among racialized minorities.

Some Indigenous and racialized participants reflected on past and contemporary events of medical harm, colonization, and racism towards racial minority populations [50]. Participants cited the “Tuskegee experiment” [51–53], the forced sterilization of Indigenous women [54], and other events to show the continuous impact on vaccine information engagement. Indeed, investigations and research have demonstrated Indigenous peoples are differently treated by the healthcare system [55], including coerced sterilization and medical mistreatment during childbirth [56–58]. In 1933, Indigenous children in Canada became subjects of tuberculosis vaccine trials without their parent’s consent, which may provide context to some vaccination concerns among Indigenous peoples [59]. This finding highlights the ongoing relevance of systemic racism and colonialism in vaccine information engagement and utilization despite the common historicization of recent and contemporary events of systemic inequities in public discourse. Historicizing unfair treatments and past harms to minority populations can interfere with responding to systemic inequities and structural barriers as ongoing problems, which (re)produce mistrust in public health information and health systems. As information is a crucial factor driving health behaviors, there is a need for health systems and authorities to acknowledge ongoing systemic racism, and to make continuous efforts towards implementing anti-racist and culturally appropriate strategies to mitigate systemic inequities and slowly earn trust. For instance, authoritative agencies should share public health information in ways that highlight awareness of existing systemic barriers and make an effort to minimize them before launching vaccination campaigns. There is a need to continuously adapt policy, terminology, and language to disseminate public health interventions and campaigns that prioritize the needs of minority populations [60]. These strategies are crucial because “one-size-fits-all” campaigns infused with colonial legacies and policies perpetuate systemic racism and inequities, which provides grounds for mistrust and vaccine refusal [47].

In Canada, prioritization of vaccine rollout focused on early access to those perceived as higher risk from the impacts of COVID-19 disease, including those with pre-existing health conditions. Some parents faced barriers in accessing relevant and credible information and challenges in discerning accurate information specific to their own and/or their children’s health conditions. Despite the urgency for protection against COVID-19, some participants were uncertain about adverse effects specific to their health conditions as reasons for the delay. This finding raises concerns because gaps in readily available and relevant information caused parents to feel unsure and frustrated, ultimately leading to delayed or refused vaccination. Those who refused vaccination often had the additional responsibility of caregiving for their children at home to protect them from COVID-19. This finding suggests further challenges in accessing information for populations who face additional barriers to trust due to systemic racism in healthcare. In an “infodemic” in which information is overabundant, great responsibility lies in the public to discern relevant, credible, and accurate information from misinformation [30]. In the Canadian healthcare context, in which patients often need to advocate for themselves, it is crucial that prioritized high-risk groups, such as those with health conditions, have access to patient-specific, relevant, and evidence-based information to guide decision-making about their vaccination.

Despite prioritization of those at high risk, our findings indicate that some populations felt excluded from consideration. Prioritization should focus on early access to life-saving vaccines for essential workers and people in high-transmission settings [61]. Yet, many workers at high risk of exposure to COVID-19 were excluded from prioritization. Public health policy should consider essential workers outside of healthcare settings who may live or work in environments that put individuals at high risk of transmission of COVID-19 [61, 62]. Throughout the pandemic, low-income essential workers, many of whom are racialized minorities, also faced challenges with customers who had the privilege of choosing to follow or not follow public health measures [63]. Essential worker participants not only faced heightened exposure to COVID-19 but often also emotional and physical abuse, which their limited personal protective equipment could not shield them from. Participants described being called racial slurs and horrific experiences of being spat at for enforcing public health and safety measures. Racialized minorities are at greater risk of stigma and emotional abuse, as evident in the rising hate crimes toward racialized people throughout the pandemic [48, 64]. Essential workers would benefit from prioritization in accessing COVID-19 vaccines and personal protective equipment, as well as access to resources to protect them from abuse on the front lines.

While public health mandates motivated COVID-19 vaccination among some participants, findings from our study also highlighted the failure of authoritative agencies to ensure that private companies and their employees adhered to public health measures. For instance, Caleb, who worked for a private company, described observing sick people infect colleagues at work and frustration for not being prioritized for early vaccination. Caleb’s experience sheds light on private companies’ interests in protecting capital at the cost of their workers, even during public health crises. We suggest that authoritative agencies devise strategies to monitor and ensure safety protocols, including dissemination of public health information are adhered to by private organizations and minimize potential exploitation of workers whose identities and social locations may not give them the fortune to forfeit working under unsafe conditions in future health crises. Failure to enforce adherence to public health measures and information, especially among private companies, compounds risks and responsibilities to deprioritized essential workers who worked during the pandemic to financially sustain their families to navigate how to protect themselves and their families from COVID-19 infection.

### Strengths and Limitations

A strength of this study is the use of an intersectional lens to understand systemic inequities that shaped COVID-19 information use and vaccine decision-making among diverse parents and their children in Canada. An additional strength of this study is the attention to social privilege alongside disadvantage. The analysis of data benefited from the diverse perspectives of research team members, from various ethnic, racial, cultural, educational, and disciplinary backgrounds. In terms of study limitations, we aimed to maximize diversity among our participants, yet some groups were underrepresented due to our recruitment strategy through an online survey, including those with irregular internet access and those with substantial English and French language barriers. We attempted to recruit participants only from the middle of the spectrum of vaccine hesitancy, but vaccine attitudes are dynamic. Thus, during the interviews, some participants were adamantly opposed or strongly supportive of vaccination. Interviews were designed to explore participants’ experiences openly and did not target specific intersections, but it is not possible for anyone to provide a full account of their experiences during an interview.

## Conclusions and Recommendations

In this study, we utilized an intersectionality-informed and feminist CDA to highlight the inequities related to information engagement and vaccination decision-making among ethnically diverse parents for themselves and their children in Canada. The use of an intersectional lens in this study aided in illuminating the constellation of intersecting inequities shared by participants, laying bare how public discourse historicization of past and contemporary events of colonialism and racism impedes health information engagement. Racialized minorities shared accounts of historical and ongoing systemic racism as a barrier to trusting and finding COVID-19 vaccine information while some participants discussed how their social privileges led to (non)compliance with public health safety measures. For some racialized participants, COVID-19 vaccine decision-making was situated within their socio-historical contexts and ongoing marginalized experiences, drawing attention to a critical need for health policy tools and program development frameworks that advance equity, restore trust, and improve access to resources. Equitable access to health and social resources, including information, is a crucial step towards empowering minority populations and reducing power differentials that perpetuate systemic inequities and oppression. In this light, we call for the adoption of transformative health policy frameworks informed by intersectionality theory to foreground and draw awareness to the complex socio-historical, political, and economic root causes of health and social problems at public policy, institutional, and structural levels. Fostering partnerships with trusted leaders and/or healthcare workers from racialized communities may help rebuild trust. A sincere partnership to intertwine the ideologies and priorities of diverse communities, health professionals and public health agencies can put forward a collective and united agenda that supports culturally appropriate health information messaging tools. Such information tools will benefit from the strengths of cultural norms and social values that are free from harmful and stigmatized language to prevent retriggering and quell fears of repeated mistreatments with the healthcare system within marginalized communities of interest. Future public health measures must consider protecting essential workers on the front line through readily available personal protective equipment and support to manage forms of abuse they may experience. Healthcare providers can use hospital visits as opportunities to discuss vaccination information specific to patients’ health conditions and provide evidence-based information to guide parents’ vaccination decision-making. Overall, our findings identify the need for the Canadian healthcare system at large to continuously implement strategies to restore trust with Indigenous and racialized populations.

## Data Availability

Data and study materials are not deposited in any public or research repository to protect participants’ confidentiality and privacy.

## Appendix

**Table 3:** Participants’ characteristics (*N* = 48)

Pseudonym	Gender	Age	Province	Reported ethnicity	Chronic health condition	Education	Child(ren)’s health condition
Ahmed	Man	52	ON	South Asian	Hypertension	Post-graduate degree	None mentioned
Aiyana	Woman	34	SK	Indigenous	None mentioned	Bachelor’s degree	None mentioned
Alice	Woman	42	ON	White	Type 2 diabetes; severe lung problem	High school diploma or equivalent	None mentioned
Anais	Woman	45	QC	White	None mentioned	Non-university certificate	One child lives with epilepsy
Andre	Man	42	QC	White	None mentioned	University certificate or bachelors	None mentioned
Antoine	Man	43	QC	Caucasian; South European	None mentioned	University certificate or bachelors	None mentioned
Asher	Man	51	ON	Southeast Asian	Health Issues (unspecified)	Bachelor’s degree	None mentioned
Aurelie	Woman	51	QC	Spanish	Immunocompromised (undergoing chemotherapy)	University certificate or bachelor’s	None mentioned
Beth	Woman	50	ON	White	None mentioned	University certificate or bachelor’s	None mentioned
Brenda	Woman	39	ON	Latin/South American	None mentioned	College, CEGEP or other non-university trade certificate, or diploma	None mentioned
Caleb	Man	52	AB	Jewish	None mentioned	University certificate or diploma below bachelor’s level	None mentioned
Calina	Woman	34	MB	Filipina	None mentioned	Bachelor’s degree	Non-mentioned
Celine	Woman	45	AB	White	None mentioned	University certificate or more than a bachelor’s	Son: Asthma Daughter: Anxiety and depression
Clove	Woman	49	QC	White	None mentioned	University certificate or bachelor’s	None mentioned
Dymond	Woman	49	ON	Jamaican Canadian	None mentioned	College, CEGEP or other non-university trade certificate, or diploma	None mentioned
Elias	Man	44	BC	East Asian	Respiratory issue	Bachelor’s degree	None mentioned
Esmond	Man	34	ON	African Canadian	Severe reaction from previous flu vaccine	Bachelor’s degree	None mentioned
Ike	Man	52	PEI	White	None mentioned	University certificate or bachelor’s or more than bachelor’s	Daughter has pneumonia
Jasper	Woman	47	BC	Chinese	None mentioned	Post-graduate degree above bachelor’s level	None mentioned
Kadie	Woman	52	MB	Metis	None mentioned	High school diploma or equivalent	Son has special needs and asthma
Kasey	Woman	49	AB	Chinese	None mentioned	Bachelor’s degree	None mentioned
Kiko	Man	50	ON	South Asian	None mentioned	Post-graduate degree	None mentioned
Leonard	Man	50	ON	Black	None mentioned	Bachelor’s degree	None mentioned
Louis	Man	57	QC	White	None mentioned	< High school	None mentioned
Madiba	Man	44	ON	South Asian	None mentioned	Bachelor’s degree	None mentioned
Marilou	Woman	51	BC	Chinese Canadian	None mentioned	High school diploma or equivalent	None mentioned
Marine	Woman	49	QC	White	Immunocompromised, psoriatic arthritis, fibromyalgia	Non-university certificate	None mentioned
Mary	Woman	39	SK	Filipino	None mentioned	College, or other non-university certificate, trade, diploma	None mentioned
Mishy	Woman	62	QC	Canadian	None mentioned	Non-university certificate	Special child; hyperactive
Myrcella	Woman	47	QC	Black (Caribbean)	None mentioned	College, or other non-university certificate, trade, diploma	None mentioned
Neila	Woman	49	BC	Venezuelan (Latin)	None mentioned	Post-graduate degree above bachelor’s level	None mentioned
Olivia	Woman	49	ON	South Asian	Asthmatic, living with obesity; allergic to penicillin, sulfur, and aspirin	Post-graduate degree above bachelor’s level	Hypothyroidism
Rachel	Two-Spirit	51	BC	First Nation	None mentioned	College, or other non-university certificate, trade, diploma	None mentioned
Rexford	Man	43	AB	Filipino	None mentioned	Bachelor’s degree	None mentioned
Sabina	Woman	44	ON	South Asian	None mentioned	Bachelor’s degree	None mentioned
Shakira	Woman	37	BC	First Nation; German	Immunocompromised (cannot take certain antibiotics)	High school diploma or equivalent	One child has disability due to autism
Shayla	Woman	35	ON	Indigenous	None mentioned	High school diploma or equivalent	None mentioned
Silvia	Woman	35	ON	Asian	None mentioned	Bachelor’s degree	None mentioned
Simran	Woman	37	ON	South Asian	None mentioned	Post-graduate degree above bachelor’s level	None mentioned
Steven	Man	40	BC	First Nation	Has a disability	College, or other non-university certificate, trade, diploma	None mentioned
Sylvie	Woman	37	QC	Caucasian	None mentioned	University certificate or bachelor’s	Youngest child has disabilities
Talata	Woman	48	QC	Arabic/West Asian/North African	None mentioned	Post-graduate degree above bachelor’s level	None mentioned
Theo	Man	53	QC	White	None mentioned	University certificate or bachelor’s	None mentioned
Vicky	Woman	47	AB	Metis	None mentioned	University certificate or bachelor’s	None mentioned
Winnie	Woman	43	BC	First Nation	None mentioned	High school diploma or equivalent	None mentioned
Youssef	Man	54	QC	Berber	None mentioned	University certificate or bachelor’s	None mentioned
Zara	Woman	49	AB	South Asian -Indian	None mentioned	Bachelor’s degree	None mentioned
Zoe	Woman	33	ON	White (German)	None mentioned	Bachelor’s degree	None mentioned

## Footnotes

1 The participant is referring to the ‘United States Public Health Service Untreated Syphilis Study at Tuskegee’ where researchers unethically engaged participants in the predominantly Black city of Tuskegee in a study about the natural history of untreated syphilis without providing informed consent and depriving them of treatment. (Centers for Disease Control and Prevention. *The United States Public Health Service Untreated Syphilis Study at Tuskegee.* Available from: https://www.cdc.gov/tuskegee/about.html).

## Code Availability

The corresponding author may be contacted for access to codes.
